# Nitrogen Metabolism and Growth Enhancement in Tomato Plants Challenged with *Trichoderma harzianum* Expressing the *Aspergillus nidulans* Acetamidase *amdS* Gene

**DOI:** 10.3389/fmicb.2016.01182

**Published:** 2016-08-03

**Authors:** Sara Domínguez, M. Belén Rubio, Rosa E. Cardoza, Santiago Gutiérrez, Carlos Nicolás, Wagner Bettiol, Rosa Hermosa, Enrique Monte

**Affiliations:** ^1^Department of Microbiology and Genetics, Spanish-Portuguese Centre for Agricultural Research, University of SalamancaSalamanca, Spain; ^2^Area of Microbiology, University School of Agricultural Engineering, University of LeonPonferrada, Spain; ^3^Department of Botany and Plant Physiology, Spanish-Portuguese Centre for Agricultural Research, University of SalamancaSalamanca, Spain; ^4^Embrapa EnvironmentJaguariúna, Brazil

**Keywords:** biocontrol, heterologous expression, amide hydrolase, GeneChip tomato genome array, plant growth, plant defense

## Abstract

*Trichoderma* is a fungal genus that includes species that are currently being used as biological control agents and/or as biofertilizers. In addition to the direct application of *Trichoderma* spp. as biocontrol agents in plant protection, recent studies have focused on the beneficial responses exerted on plants, stimulating the growth, activating the defenses, and/or improving nutrient uptake. The *amdS* gene, encoding an acetamidase of *Aspergillus*, has been used as a selectable marker for the transformation of filamentous fungi, including *Trichoderma* spp., but the physiological effects of the introduction of this gene into the genome of these microorganisms still remains unexplored. No evidence of *amdS* orthologous genes has been detected within the *Trichoderma* spp. genomes and the *amdS* heterologous expression in *Trichoderma harzianum* T34 did not affect the growth of this fungus in media lacking acetamide. However, it did confer the ability for the fungus to use this amide as a nitrogen source. Although a similar antagonistic behavior was observed for T34 and *amdS* transformants in dual cultures against *Rhizoctonia solani, Botrytis cinerea*, and *Fusarium oxysporum*, a significantly higher antifungal activity was detected in *amdS* transformants against *F. oxysporum*, compared to that of T34, in membrane assays on media lacking acetamide. In *Trichoderma*-tomato interaction assays, *amdS* transformants were able to promote plant growth to a greater extent than the wild-type T34, although compared with this strain the transformants showed similar capability to colonize tomato roots. Gene expression patterns from aerial parts of 3-week-old tomato plants treated with T34 and the *amdS* transformants have also been investigated using *GeneChip Tomato Genome Arrays*. The downregulation of defense genes and the upregulation of carbon and nitrogen metabolism genes observed in the microarrays were accompanied by (i) enhanced growth, (ii) increased carbon and nitrogen levels, and (iii) a higher sensitivity to *B. cinerea* infections in plants treated with *amdS* transformants as detected in greenhouse assays. These observations suggest that the increased plant development promoted by the *amdS* transformants was at expense of defenses.

## Introduction

The fungal genus *Trichoderma* includes species that have the ability to antagonize, parasitize, or even kill other fungi (Lorito et al., 2010; Druzhinina et al., 2011). Some strains of *Trichoderma* are used as biocontrol agents in agriculture and can establish themselves in the plant rhizosphere, stimulate plant growth, and elicit plant defense reactions against pathogens (Hermosa et al., 2012). It has also been observed that selected *Trichoderma* strains can improve plant nutrient uptake (Yedidia et al., 2001). The molecular mechanisms involved in plant responses to *Trichoderma* root colonization have been explored through transcriptomic (Bailey et al., 2006; Alfano et al., 2007; Bae et al., 2011; Morán-Diez et al., 2012), proteomic (Marra et al., 2006; Segarra et al., 2007; Shoresh and Harman, 2008), and both proteomic and metabolomic (Bae et al., 2011; Brotman et al., 2012) approaches. The results of these studies have confirmed the previous findings that *Trichoderma* is important for regulating many genes involved in plant defense against biotic and abiotic stresses, or for increasing the plant basal metabolism (i.e., photosynthetic rate or respiratory activities). *Trichoderma* is able to control a broad range of plant pathogens through elicitation of induced systemic resistance (ISR) or localized resistance (Shoresh et al., 2010). Root colonization with *Trichoderma* primes leaf tissues for enhanced activation of jasmonic acid (JA)-regulated defense responses leading to a higher resistance to necrotrophic pathogens (Tucci et al., 2011; Hermosa et al., 2012; Mathys et al., 2012; Martínez-Medina et al., 2013). In addition to indol acetic acid (IAA) (Contreras-Cornejo et al., 2009), *Trichoderma* spp. produce enzymes and metabolites able to change ethylene levels in the plant (Viterbo et al., 2010) and to modify root architecture (Rubio et al., 2009, 2012; Samolski et al., 2012; Malmierca et al., 2015), achieving a more efficient nutrient uptake. A recent study has shown that 6-pentyl-2*H*-pyran-2-one, the major volatile organic compound produced by *Trichoderma* spp., promotes plant growth and regulates root architecture through a mechanism involving components of auxin transport and signaling and the ethylene-response modulator EIN2 (Garnica-Vergara et al., 2015). Since modern agriculture is a major cause of environmental pollution, including large-scale nitrogen- and phosphorus-induced environmental changes, the additional amounts of nitrogen activated by humans must be curtailed so as to avoid disastrous consequences for humanity (Rockström et al., 2009). It has been hypothesized that the use of *Trichoderma* could reduce the quantities of nitrogen fertilizers used about 40–50% without a reduction in crop yield (Harman, 2011). This plant enhancer trait associated to *Trichoderma* would have more impact in agriculture than its common use as biocontrol agent (Shoresh et al., 2010).

In a previous study describing the cloning and characterization of the transcription factor *Thctf1* of *Trichoderma harzianum* T34 we produced null mutants for this gene (Rubio et al., 2009). To generate such mutants, a disruption cassette for *Thctf1* was constructed into the Promega pGEM-T vector using the *amdS* gene of *Aspergillus nidulans*, which encodes an enzyme with aliphatic amidase activity, and used as a selectable marker for *T. harzianum* transformation. When greenhouse tests were performed with tomato seeds coated with the *T. harzianum* wild-type strain, increased root development was observed, as could be expected due to *Trichoderma*' s ability to promote plant growth (Shoresh et al., 2010; Hermosa et al., 2013). Furthermore, the increase in root size was much more evident with the *Thctf1* null mutants of *T. harzianum*. Although it might be assumed that these differences were attributed to the disruption of *Thctf1*, an ectopic integration control strain included in the greenhouse assays provoked a similar increase in root elongation to that observed for the null mutants. These results indicated that this beneficial effect to the plant was not due to the interruption of *Thctf1* but to the expression of the *amdS* gene of *A. nidulans* included within the disruption cassette. The *A. nidulans amdS* gene was used as a selectable marker for the transformation of filamentous fungi as it is a non-antibiotic marker that is only present in a few fungal species, and because the transformants carrying the *amd*S gene could be selected using acetamide as the sole carbon or nitrogen source (Penttilä et al., 1987).

Amidases (acylamide amidohydrolases EC 3.5.1.4) are hydrolytic enzymes able to catalyze the conversion of carboxylic amides to the corresponding carboxylic acid and ammonia. Amidases can exhibit activity with aliphatic amides (acetamide, acrylamide, and propionamide), with mid-chain aliphatic amides coupled with nitrile hydratases (propionamide, isobutyramide, valeramide, and hexanoamide), and with aromatic amides (benzamide, phenylacetamide, indoleacetamide; Fournand and Arnaud, 2001). As ammonia producers, amidases participate in nitrogen metabolism and apart from amidohydrolase activity they have been shown to be involved in one of the several pathways for the formation of IAA (Lehmann et al., 2010). It has long been known that the *amdS* gene of *A. nidulans* codes for an acetamidase enzyme that hydrolyses acetamide to acetate and ammonium providing carbon and nitrogen sources for fungal growth (Hynes and Pateman, 1970; Hynes, 1994).

To understand the role of acetamidase activity in the interaction of the biocontrol agent *T. harzianum* with tomato plants, *T. harzianum* T34 transformants expressing the *amdS* gene from *A. nidulans* were obtained in the present work. Transformants amdS6 and amdS122, carrying one and two copies of the *amdS* gene, respectively, were used along this study. Heterologous expression of *amdS* reproduced the acetamidase activity in *T. harzianum* and increased the ability of this fungus to promote the growth of tomato plants. The *amdS* transformants showed higher growth than the wild-type strain only when acetamide was included in the culture media. In dual culture biocontrol assays using *Rhizoctonia solani, Botrytis cinerea*, and *Fusarium oxysporum* as targets, no differences in antagonistic activity were detected between strain T34 and *amdS* transformants. However, in membrane assays on media lacking acetamide, the transformants showed significant higher antifungal activity than strain T34 against *F. oxysporum*. In *in vivo* assays, tomato plants colonized by *amdS* transformants showed an increased availability of ammonium ions and gave rise to a significant upregulation of a set of genes involved in the control of central nitrogen metabolism of the plant. However, all of these positive effects to the plants were accompanied by an increased sensitivity to the pathogen *B. cinerea*.

## Materials and methods

### Microorganisms and tomato seeds

*Escherichia coli* DH5α was used as the host for plasmid construction and propagation. This bacterial strain was grown in Luria-Bertani (LB) broth or on LB plates, supplemented with ampicillin (100 μg/ml), X-gal (40 μg/ml), and IPTG (10 μg/ml), when required.

*T. harzianum* CECT 2413 (Spanish Type Culture Collection, Valencia, Spain), referred to here as T34 strain, was used as the wild-type strain throughout this study. T34 was used as a host in the transformation experiments with the *A. nidulans amdS* gene. All strains were propagated on potato dextrose agar (PDA, Difco Laboratories, Detroit, USA).

*F. oxysporum* f. sp. *lycopersici* CECT 2866, *R. solani* CECT 2815, and *B. cinerea* 98 (isolated from strawberry at our lab) were used as the plant pathogenic microorganisms in *in vitro* antagonism assays. *B. cinerea* was also used as a pathogen in the *in vivo* assays. These fungal strains were grown on PDA medium and were stored at −80°C in 30% glycerol (*F. oxysporum* and *B. cinerea*) and at 4°C in PDA plugs suspended in sterile water (*R. solani*).

Tomato seeds (*Solanum lycopersicum* “Marmande”) were sterilized in 2% sodium hypochlorite for 20 min and washed thoroughly in sterile distilled water before being used.

### Fungal culture conditions

For gene expression, *T. harzianum* strains were grown in potato dextrose broth (PDB, Difco Laboratories) and PDB plus 10 mM acetamide. After 48 h of incubation at 28°C and shaken at 200 rpm, mycelia were collected by filtration, thoroughly washed with sterile water, lyophilized, and kept at −80°C until RNA extraction. Three cultures were used for each condition.

For determination of growth rates, *T. harzianum* strains were inoculated at the center of Petri dishes containing water agar (WA) medium with 1.5% agar, Murashige and Skoog (MS) medium (Duchefa Biochemie, Haarlem, The Netherlands) supplemented with 1% (w/v) sucrose and 0.8% agar pH 5.7, PDA or PDA plus 10 mM acetamide, and incubated at 28°C. Six plates were used for each condition. The colony diameters were determined after 48 h of incubation. Fungal biomass was also calculated after growth in liquid media at 28°C and shaken at 200 rpm for 48 h. Mycelia from PDB and PDB plus 10 mM acetamide cultures were collected by filtration, lyophilized and their dry weights were measured. Supernatants of the *T. harzianum* liquid cultures were used for ammonium quantification.

For the tomato root colonization studies, *T. harzianum* strains were grown in minimal medium (MM, Penttilä et al., 1987) containing 2% glucose as carbon source. After 48 h of incubation at 28°C and shaken at 200 rpm, mycelia were collected by filtration and thoroughly washed with sterile water.

### DNA procedures and conventional PCR amplification

Total DNAs from fungi were extracted following the method of Raeder and Broda (1985), using mycelium collected from a PDB culture incubated at 28°C and shaken at 200 rpm for 48 h.

For colonization assay, DNA isolation was performed with a cetyltrimethylammonium bromide (CTAB) extraction method (Dellaporta et al., 1983).

For Southern analysis, 10 μg of genomic DNA were *EcoR*V-digested, electrophoresed on a 0.7% agarose gel, and transferred to a Hybond-N^+^ membrane (Amersham Biosciences AB, Uppsala, Sweden). A fragment of 2203 bp, obtained from the DNA of the pLMG::*amdS* plasmid using the primers gpd3F and cbh2 (Table S1), was labeled using the DIG High Prime kit (Roche, Penzberg, Germany), following the manufacturer' s instructions and used as probe. Hybridization, washes, and detection were carried out as previously described (Rubio et al., 2008).

Standard PCRs were accomplished using the *Taq* polymerase system (Biotools B&M Labs. S.A., Madrid, Spain), and the Expand Long Template PCR System (Roche) was used to amplify fragments larger than 2 kb, following the manufacturer' s instructions. The primers used to do this are shown in Table S1.

### Real-time PCR analysis

Gene expression was analyzed by quantitative real-time PCR. cDNA was synthesized from 2 μg of RNA, which was extracted using TRIZOL® (Invitrogen Life Technologies, Carlsbad, USA) and treated with DNase RQ1 (Promega Biotech Ibérica, Alcobendas, Spain), and then used for reverse transcription with an oligo(dT) primer with the Transcriptor First Strand cDNA Synthesis kit (Takara Inc., Tokyo, Japan), following the manufacturer' s protocol. Three five-plant sample sets from three different *in vivo* assays per condition were used (biological replicates). Aerial part from every five-plant set per condition and independent assay was pooled for RNA extraction, and the subsequent cDNA synthesis. Real-time PCR reactions were performed with a thermocycler StepOnePlus (Applied Biosystems, Foster City, USA) in a total volume of 10 μl using SYBR FAST KAPA qPCR (Biosystems, Buenos Aires, Argentina) and a final primer concentration of 100 nM each. The primer pairs used are shown in Table S1. Reactions were performed in triplicate under the following conditions: an initial denaturation step (10 min at 95°C) followed by 40 cycles of denaturation (30 s at 95°C), annealing (1 min 60°C), and extension (1 min 72°C). Ct (cycle threshold) values were calculated using the Applied Biosystems software, and transcript abundance was calculated in Microsoft Excel from Ct values and normalized to the *actin* gene signal. The slopes and efficiency for each primer pair were measured for a dilution series of pooled cDNA samples and calculated using the Applied Biosystems software (Table S1). The relative expression levels were calculated using the 2^−ΔΔCT^ method (Livak and Schmittgen, 2001).

The quantification of wild-type and *amdS* transformant DNA from colonized tomato roots was also performed by quantitative real-time PCR. The reaction mixes and real-time PCR conditions were the same described above and DNA from the *Trichoderma*-root interaction was used as template. Specific primers for the amplification of a fragment of the *Trichoderma actin* gene, and the tomato *actin* gene were used (Table S1). The Ct values were calculated and the amount of fungal DNA was estimated using standard curves. Values were normalized to the amount of tomato DNA in the samples. Each sample was tested in triplicate.

### Plasmid constructions and *Trichoderma* transformation

The plasmid pLMG::*amdS* (Figure S1), carrying the transformation cassette was constructed. To do this, the plasmid p3SR2 (Kelly and Hynes, 1985) was first used as a template to PCR-amplify the *amdS* gene of *A. nidulans*, which encodes the acetamidase, using the primers amdS-NT and amdS-CT (Table S1). Then, the pLMG plasmid, which contained the *gpdA* (glyceraldehyde-3-phosphate dehydrogenase A) gene promoter from *A. nidulans* and the *cbh2* (cellobiohydrolase II) terminator region from *Trichoderma reesei* was digested with *Xba*I, treated with a Klenow fragment, and finally ligated to the PCR-amplified *amdS* gene to obtain the final vector pLMG::*amdS* of 6484 bp (Figure S1). This plasmid was linearized with *Eco*RI and used to transform protoplast of *T. harzianum* T34. Protoplast preparation, transformation, and transformant stabilization were carried out according to Cardoza et al. (2006). Transformants were identified by growth on selective medium containing 10 mM acetamide as sole nitrogen source.

### Ammonium quantification

Ammonium concentration was determined using the Spectroquant® Ammonium Test (Merck, Madrid, Spain) kit based on the phenol-hypochloride method, following to the instructions of manufactures. The ammonium liberated was determined spectrophotometrically using different concentrations of NH_4_Cl in a range of 0–250 μM as standards.

### *In vitro* antifungal assays

Confrontation assays (dual cultures) on PDA plates between *T. harzianum* strains and the pathogens *F. oxysporum, R. solani*, and *B. cinerea* were carried out in triplicate as previously described (Rubio et al., 2009). The dual cultures were examined after 6 days of incubation.

Growth assays on cellophane and dialysis membranes were carried out on PDA and PDA plus 10 mM acetamide plates in triplicate, as previously described (Rubio et al., 2009). Briefly, a five-millimeter-diameter PDA plug of T34 or transformants (amdS6 and amdS122) was placed at the center of a petri plate containing PDA or PDA plus acetamide medium, on a cellophane sheet, or on a 10-kDa-cutoff dialysis membrane. After 2 days of incubation at 28°C, the membrane was removed from the plate, and a single 5-mm diameter mycelial plug of the pathogen was placed at the center of the plate. In parallel, the pathogen was grown on PDA or PDA plus acetamide (control). This assay was carried out in triplicate for every *T. harzianum* strain, pathogen, medium, and membrane. Growth diameters were measured after 72 h for *R. solani* and *B. cinerea* and 6 days for *F. oxysporum*. Results are expressed as the percentage of growth inhibition of each pathogen by each *T. harzianum* strain with respect to the mean colony diameters of each pathogen grown alone.

The extracellular antifungal activity of *T. harzianum* strains against *F. oxysporum* and *B. cinerea* was tested as previously described (Pérez et al., 2015), using 100 μl of filter-sterilized unboiled supernatants from 48 h-PDB and 48 h-PDB plus 10 mM acetamide cultures. Wells containing 100 μl of every medium were used as controls. *F. oxysporum* and *B. cinerea* growth was determined at 0, 24, and 48 h by measuring optical density at 595 nm using a Sunrise microtiter plate reader (Tecan Ibérica, Barcelona, Spain). Each test was performed in sextuplicate.

### *In planta* experiments

An *in vitro* assay was carried out to analyze the effect of *T. harzianum* strains on tomato seedlings. This assay was performed as previously described (Rubio et al., 2012). Briefly, inocula of 1 × 10^6^ *Trichoderma* spores (10 μl of an aqueous suspension containing 1 × 10^8^ spores ml^−1^) were placed on the MS medium plates, supplemented with 1% sucrose and 0.8% agar, pH 5.7, at the opposite end to where 5-day-old germinated tomato seedlings (five seedlings per plate) were located. Plates were cultured in a growth chamber under conditions of 40% humidity, 24°C, and a 16 h light/8 h dark photoperiod. MS plates containing only tomato seedlings, without *Trichoderma* spores, were used as controls. Experiments were performed in triplicate and measurements of stem length were taken at 4 days after *Trichoderma* inoculation.

To perform the tomato root colonization test, 3-week-old plants were cultured in a 500-ml Erlenmeyer flasks containing 400 ml of liquid MS medium inoculated with mycelium of *Trichoderma* strain (T34, amdS6, or amdS122), which were incubated in an orbital shaker at 80 rpm and 25°C. In parallel, tomato plants were cultured in MS medium without fungus (control). Mycelia were obtained from 48-h-old cultures of the strains in 400 ml of MM containing 2% glucose as indicated above. After 20 h of *Trichoderma*-plant interaction or control culture, roots from five plants per treatment were collected, washed with distilled water, frozen, lyophilized, and kept at −80°C until total DNA extraction. Three independent tomato-*Trichoderma* co-culture experiments were carried out for each strain.

The ability of *T. harzianum* strains to promote the growth of tomato plants was also evaluated in an *in vivo* assay. Sterilized tomato seeds were coated, as previously described (Pérez et al., 2015), with an aqueous suspension containing 2 × 10^8^ spores ml^−1^ of *T. harzianum* T34, amdS6, or amdS122 (1 ml of spore suspension/30 seeds) and then air-dried in an open Petri plate overnight under a laminar flow hood. Treated tomato seeds were sown in pots containing commercial loamy field soil, previously autoclaved at 121°C, for 1 h on two successive days. Pots with untreated tomato seeds were used as controls. The pots were cared for in a greenhouse at 22 ± 4°C, and watered as needed. The measurements of stem and main root lengths from 24 tomato plants for each treatment were taken after 3 weeks. Aerial parts were collected from tomato plants at this time, frozen, and kept at −80°C for microarray experiments. Aerial parts were collected from 25 tomato plants, dried at 65°C for 2 days in five batches of five plants each, powered and submitted to the CEBAS Center (Murcia, Spain) for carbon and nitrogen determination.

Sensitivity of 3-week-old tomato plants, *T. harzianum*-treated or untreated (control), to *B. cinerea* was evaluated in an *in vivo* assay as previously described (Pérez et al., 2015), with some modifications. Two leaves from each plant were inoculated in a spot using 10 μl of a germination solution (20 mM glucose and 20 mM potassium phosphate) containing 2000 *B. cinerea* conidia per spot. Three days after *B. cinerea*-infection, leaves were detached and photographed, and necrotic leaf area was calculated using ImageJ free software. Six plants were analyzed for every condition. Data are presented as mean of necrotic foliar area percentage with standard deviation.

### Tomato microarray experiments

Total RNA was obtained from aerial parts of 3-week-old tomato plants using the NucleoSpin® RNA Plant kit (Macherey-Nagel GmbH and Co. KG, Düren, Germany), and was purified using the RNeasy MinElute Cleanup kit (Qiagen, Hilden, Germany), following the manufacturer' s instructions. cDNAs were synthesized from 1 μg of total RNA using the Transplex Whole Transcriptome Amplification kit (Sigma-Aldrich Quimica S.L., Madrid, Spain), were labeled using the GeneChip IVT Labeling kit (Affymetrix, Santa Clara, USA) and subsequently used for hybridization to Affymetrix GeneChip Tomato Genome Arrays. Twelve tomato chips at a reason of three chips per each condition considered [untreated plant (control) and plants challenged with T34, amdS6, or amdS122] were used. Every chip was hybridized with cDNA prepared from total RNA extracted from five pooled plants.

Digitalization of the fluorescent signals emitted after the hybridization was performed using the Gene Array Reader (Affymetrix) and the GCOS and Desktop Mining Solution (Micro DB 3.0, Data MiningTool 3.0) programs. Data were deposited in the GEO database with accession number GSE76332. A Robust Multichip Average (RMA) convolution model was applied for background correction, and the corrected probe intensities were then normalized using a quantile-based normalization procedure, as performed by Irizarry et al. (2003). To identify probe sets showing a significant difference in expression level, a multi-class Significance Analysis of Microarray (SAM) test was carried out on the expression values using a False Discovery Rate (FDR) of 0.10. The analysis was performed using the Gene Spring GX program through the R software. Transcripts showing significantly differential expression [fold-change (FC) ≥ 2 and FDR 0.10] were annotated according to gene ontology (GO) terms (Ashburner et al., 2000), which were based on the BLAST definitions obtained after applying an *E* < 10^−10^ level.

### Statistical analyses

Analysis of variance (ANOVA) was conducted with SPSS v.19 software (SPSS Inc., Chicago, USA). Pairwise comparisons were made using the Tukey' s test at *P* < 0.05, except for microarray data that were analyzed as already described above.

## Results

### Expression of *A. nidulans amdS* gene in *T. harzianum* and characterization of *amdS* transformants

The strain *T. harzianum* T34 was transformed with the pLMG::*amdS* plasmid that had been previously linearized with the endonuclease *Eco*RI to facilitate its later introduction into the recipient strain. Transformants were selected in medium containing acetamide as the sole nitrogen source and after three selection culture rounds, 45 mitotically stable transformants were obtained and checked by PCR. A 1743-bp PCR product was observed in 15 out of the 45 transformants, but not in the wild-type strain, using the primers gpd3F and amdS-3. Monosporic isolates were obtained for these 15 transformants and the integration of the transformation cassette was analyzed by Southern blot using a 2203 bp fragment from the pLMG::*amdS* plasmid as a probe. The probe did not hybridize to wild-type T34 DNA and the different integration patterns observed among the 15 transformants are shown in Figure S1. Strains amdS6 and amdS122, including one and two copies of the transformation cassette, respectively, were chosen for further studies.

**Figure 1:**
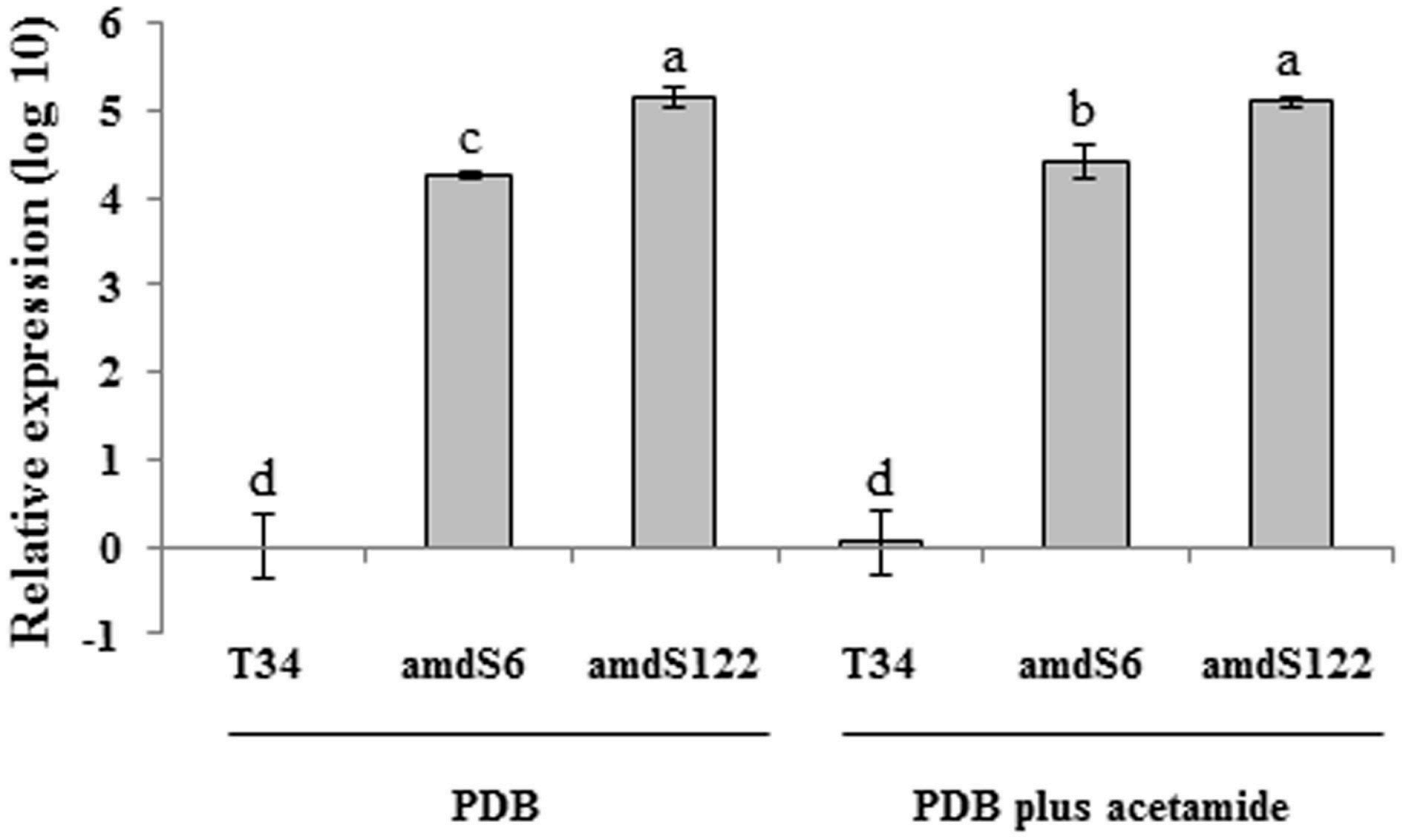
**Quantification of *amdS* transcript in *T. harzianum* wild-type (T34) and two transformants (amdS6 and amdS122) by real-time PCR**. The experiment was carried out with mycelia grown for 48 h on PDB or PDB plus 10 mM acetamide. Values correspond to relative measurement against the *amdS* transcript in T34 grown on PDB medium (2^−ΔΔCt^ = 1). The relative expression measurements in the *Y* axis are indicated in a logarithmic scale. *T. harzianum* T34 *actin* was used as an internal reference gene. Bars represent the standard deviation of the mean of three replicates. The levels of expression were tested using one-way analysis of variance (ANOVA) followed by Tukey' s test. Different letters represent significative differences (*P* < 0.05).

Both transformants and the wild-type were grown on WA, MS, PDA, and PDA plus acetamide plates. No growth differences were observed among the three strains on WA, MS, or PDA whereas *T. harzianum* T34 exhibited a significantly lower growth rate than the *amdS* transformants on PDA supplemented with acetamide (Table 1). In addition, both transformants displayed a higher mycelium dry weight than T34 after growing in PDB supplemented with acetamide, whereas no growth differences were observed in PDB (data not shown). We compared the *amdS* expression levels of amdS6 and amdS122 grown in PDB and PDB plus acetamide with those of T34 grown under the same conditions, using the primers amdS-RT1 and amdS-RT2. Additionally, the transcript level of T34 in PDB was used as the reference condition. Both transformants showed higher transcript levels than the wild-type in the two media, with amdS122 displaying the highest expression (Figure 1). When ammonium concentration was determined in the supernatants from these two culture media, only detectable amounts were observed for transformants amdS6 and amdS122 grown in PDB plus acetamide; concentrations were determined as 2.60 ± 0.36 and 3.36 ± 0.35 mM of ammonium, respectively.

**Table 1:** ***Trichoderma* growth rate on different culture media**.

	**WA**	**MS**	**PDA**	**PDA plus acetamide**
T34	4.88 ± 0.16a	5.32 ± 0.23a	5.76 ± 0.09a	5.45 ± 0.12a
amdS6	5.0 ± 0.14a	5.28 ± 0.19a	5.85 ± 0.13a	5.89 ± 0.11b
amdS122	4.95 ± 0.19a	5.23 ± 0.12a	5.89 ± 0.011a	5.89 ± 0.15b

*Colony diameters (cm) of T. harzianum wild-type (T34), and amdS transformants (amdS6 or amdS122) were measured on plates containing WA, MS, PDA, or PDA plus 10 mM acetamide after 48 h of incubation*.

*Values are the means of six biological replicates. For each medium, values followed by different letters are significantly different (P < 0.05)*.

### Antifungal activity

The influence of the *amdS* gene in the antifungal activity of *T. harzianum* T34 was evaluated in different *in vitro* assays with the phytopathogens *F. oxysporum, B. cinerea*, and/or *R. solani* as targets. The three *T. harzianum* strains were able to inhibit the growth of the three pathogens tested in dual confrontation experiments on PDA, and no differences were observed between the wild-type and the *amdS* transformants (data not shown).

The antagonistic potential of *T. harzianum* extracellular compounds against these pathogens was evaluated using cellophane and dialysis (cellulose, cut-off 10 kDa) membrane assays on PDA and PDA plus acetamide plates. After removal of the membranes containing the *T. harzianum* mycelia, the effect of hydrolytic enzymes plus metabolites (cellophane) or only metabolites (dialysis) on the growth of the three plant pathogens was determined. The *F. oxysporum, R. solani*, and *B. cinerea* growth inhibition percentages calculated for the three *T. harzianum* strains are summarized in Table 2. All assayed *Trichoderma* strains were able to inhibit the growth of the three pathogens. Moreover, the inhibition values recorded for growth on cellophane were generally higher than those on dialysis membrane, except for *B. cinerea* and the amdS6 transformant on PDA. No differences of *B. cinerea* and *R. solani* growth inhibition were observed among the three *T. harzianum* strains in membrane PDA tests, but lower inhibition values against *F. oxysporum* were obtained for T34 than those for the two *amdS* transformants in both PDA assays. However, higher inhibition values against the three pathogens were always obtained with T34 than with *amdS* transformants when the assays were performed on PDA plus acetamide medium, although no significant differences were detected between T34 and amdS6 for *R. solani* in the dialysis membrane test.

**Table 2:** **Antifungal activity in membrane assays**.

		**PDA**		**PDA plus acetamide**	
		**Cellophane**	**Dialysis**	**Cellophane**	**Dialysis**
*R. solani*	T34	48.25 ± 8.17a	36.10 ± 7.05a	56.87 ± 3.75a	21.41 ± 3.43a
	amdS6	55.50 ± 4.10a	42.81 ± 5.06a	38.44 ± 8.17b	15.02 ± 7.34a
	amdS122	55.26 ± 3.64a	48.56 ± 6.23a	28.75 ± 7.21b	4.79 ± 1.98b
*B. cinerea*	T34	74.31 ± 7.17a	66.26 ± 2.67a	76.09 ± 3.95a	69.85 ± 0.73a
	amdS6	65.47 ± 3.22a	67.72 ± 2.97a	44.29 ± 8.28b	35.50 ± 7.99b
	amdS122	69.89 ± 2.20a	67.23 ± 0.80a	48.37 ± 5.52b	31.72 ± 7.60b
*F. oxysporum*	T34	34.62 ± 6.50b	23.74 ± 4.75b	46.03 ± 3.24a	32.79 ± 5.68a
	amdS6	50.64 ± 1.28a	40.91 ± 4.18a	35.98 ± 3.07b	15.57 ± 6.76b
	amdS122	58.55 ± 4.98a	35.86 ± 4.30a	31.59 ± 3.16b	14.75 ± 6.56b

*Percentages of growth inhibition of R. solani, B. cinerea, and F. oxysporum by T. harzianum wild type (T34), and amdS transformants (amdS6 or amdS122) grown on cellophane or dialysis membranes on PDA or PDA plus 10 mM acetamide plates*.

*Values are the means of three biological replicates. For each pathogen, type of membrane, and medium, values followed by different letters in each column are significantly different (P < 0.05)*.

**Figure 2:**
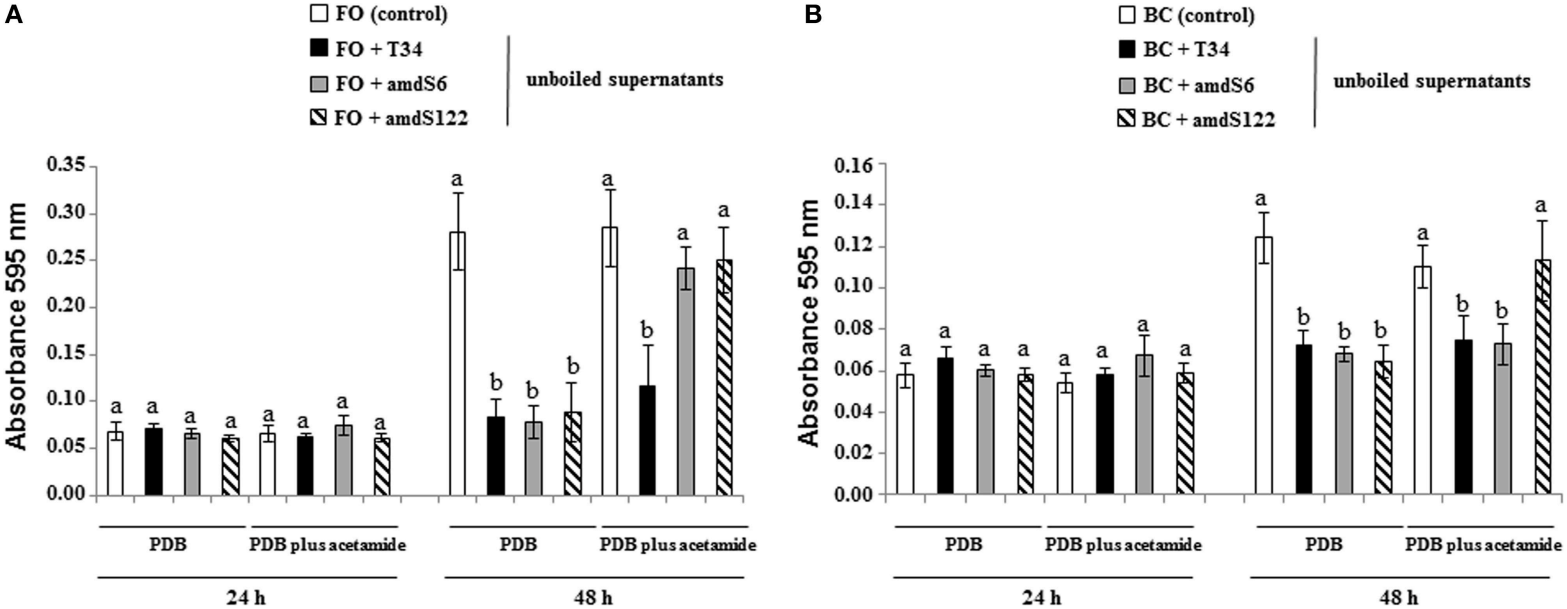
**Effect of *T. harzianum* supernatants in the growth of *F. oxysporum* (FO) (A) and *B. cinerea* (BC) (B)**. Tests were conducted without (control) or with 100 μl of filter-sterilized unboiled supernatant from a 48 h-PDB and 48 h-PDB plus 10 mM acetamide cultures of strains T34, amdS6, and amdS122. Fungal growth was determined after 28°C incubation at 24 and 48 h by measuring absorbance at 595 nm using a microtiter plate reader. Values are means of six replicates. The differences for every pathogen, culture medium and incubation time were tested using one-way analysis of variance (ANOVA) followed by Tukey' s test. Different letters represent significative differences (*P* < 0.05).

The antifungal activity of *T. harzianum* PDB or PDB plus acetamide supernatants was evaluated against *F. oxysporum* and *B. cinerea* on 96-well E plates. In this assay, the hyphal growth from conidia of the two target fungi was registered at 0, 24, and 48 h. The absorbance values recorded at 0 (data not shown) or 24 h did not show significant differences among the eight conditions assayed for each pathogen (Figure 2). PDB supernatants from the three *T. harzianum* strains had significant antifungal activity against *F. oxysporum* and *B. cinerea* at 48 h but no significant differences were observed among *Trichoderma* strains. The highest inhibition value of *F. oxysporum* was obtained from the PDB-acetamide supernatant where the wild-type strain had been previously grown. The amdS122 PDB-acetamide supernatant did not show antifungal activity against *F. oxysporum* and *B. cinerea*.

### Effect of *Trichoderma amdS* transformants on tomato plants

To compare the effect of *T. harzianum* T34 and the two *amdS* transformants on the growth of tomato seedlings, an *in vitro* assay was carried out. All *Trichoderma* strains exerted beneficial effects on the stem development, and *amdS* transformants-treated plants showed aboveground length values significantly higher than those measured in T34-treated plants (Table 3).

**Table 3:** **Effect of *Trichoderma* on tomato plant growth**.

	**Treatment**	**Control**	**T34**	**amdS6**	**amdS122**
*In vitro* assay^*^	Stem length (cm)^*^	3.91 ± 0.15a	4.36 ± 0.14b	4.86 ± 0.15c	5.12 ± 0.11c
*In vivo* assay^**^	Root length (cm)	19.10 ± 2.20a	20.70 ± 2.20b	22.30 ± 3.10c	23.50 ± 2.80c
	Stem length (cm)	9.30 ± 0.90a	10.20 ± 0.90b	11.50 ± 0.80c	12.30 ± 0.70d
	Nitrogen (mg/plant)	9.59 ± 0.47a	12.54 ± 0.78b	14.69 ± 1.40c	16.18 ± 9.9c
	Carbon (mg/plant)	69.44 ± 7.32a	84.76 ± 5.27b	98.65 ± 7.05c	107.19 ± 9.29c

*Tomato stem and root length values, and total amount of nitrogen and carbon in aerial part of plants treated with T. harzianum wild-type (T34) and amdS transformants (amdS6 or amdS122)*.

* *For the in vitro assay, 5-day-old germinated tomato seedlings grown on MS medium, supplemented with 1% sucrose and 0.8% agar, pH 5.7, were inoculated with water, T34, amdS6, or amdS122, and stem length values were taken 4 days after Trichoderma inoculation. Values are the means of 15 measurements from three biological replicates*.

** *For the in vivo assay, root and stem length values (cm), and nitrogen and carbon amounts (mg/plant) were measured in 3-week-old tomato plants developed from untreated (control), T34-treated, amdS6-treated, or amdS122-treated seeds. Root and stem length data are the means of 24 measurements and similar results were observed in three independent experiments. Nitrogen and carbon values are the means of 25 measurements. In all assays, values followed by different letters in each line are significantly different (P < 0.05)*.

The effect of *amdS* expression in *T. harzianum* regarding the ability of this fungus to colonize plant roots was evaluated by real-time PCR using the two *amdS* transformants and the wild-type strain. No significant differences among the ratios of fungal and plant DNA amounts were detected (Table 4).

**Table 4:** **Colonization of tomato roots by *Trichoderma harzianum* wild-type T34 and the *amdS* transformants**.

	***Trichoderma* a*ctin***				**Tomato *actin***				
**Condition**	**Ct**	**SD**	Qty^*^	**SD**	**Ct**	**SD**	Qty^**^	**SD**	Ratio^***^
T34	20.77	1.33	2.04	0.42	29.15	0.85	0.88	0.31	2.45a
amdS6	20.30	0.57	2.19	0.18	28.67	0.66	1.06	0.24	2.12a
amdS122	20.17	1.07	2.23	0.34	28.80	1.40	1.01	0.52	2.71a

*Fungal DNA present on the tomato roots 20 h after inoculation was quantified by real-time PCR*.

* *Quantity of fungal DNA (ng) referred to Trichoderma actin gene*.

** *Quantity of plant DNA (ng) referred to tomato actin gene*.

*** *Proportion of fungal DNA vs. plant DNA. Values are means of three biological replicates with the corresponding standard deviation. Values followed by the same letter are not significantly different (P < 0.05)*.

Three-week-old tomato plants previously seed-coated with an aqueous solution (control) or treated with conidia of T34, amdS6, or amdS122 were also evaluated in an *in vivo* assay. There were significant differences in stem and root lengths of 3-week-old tomato plants between the treatments with T34 and *amdS* transformants (Table 3), with the highest sizes being observed in tomato plants challenged with the two *amdS* transformants. Similar results were observed in three independent experiments.

**Figure 3:**
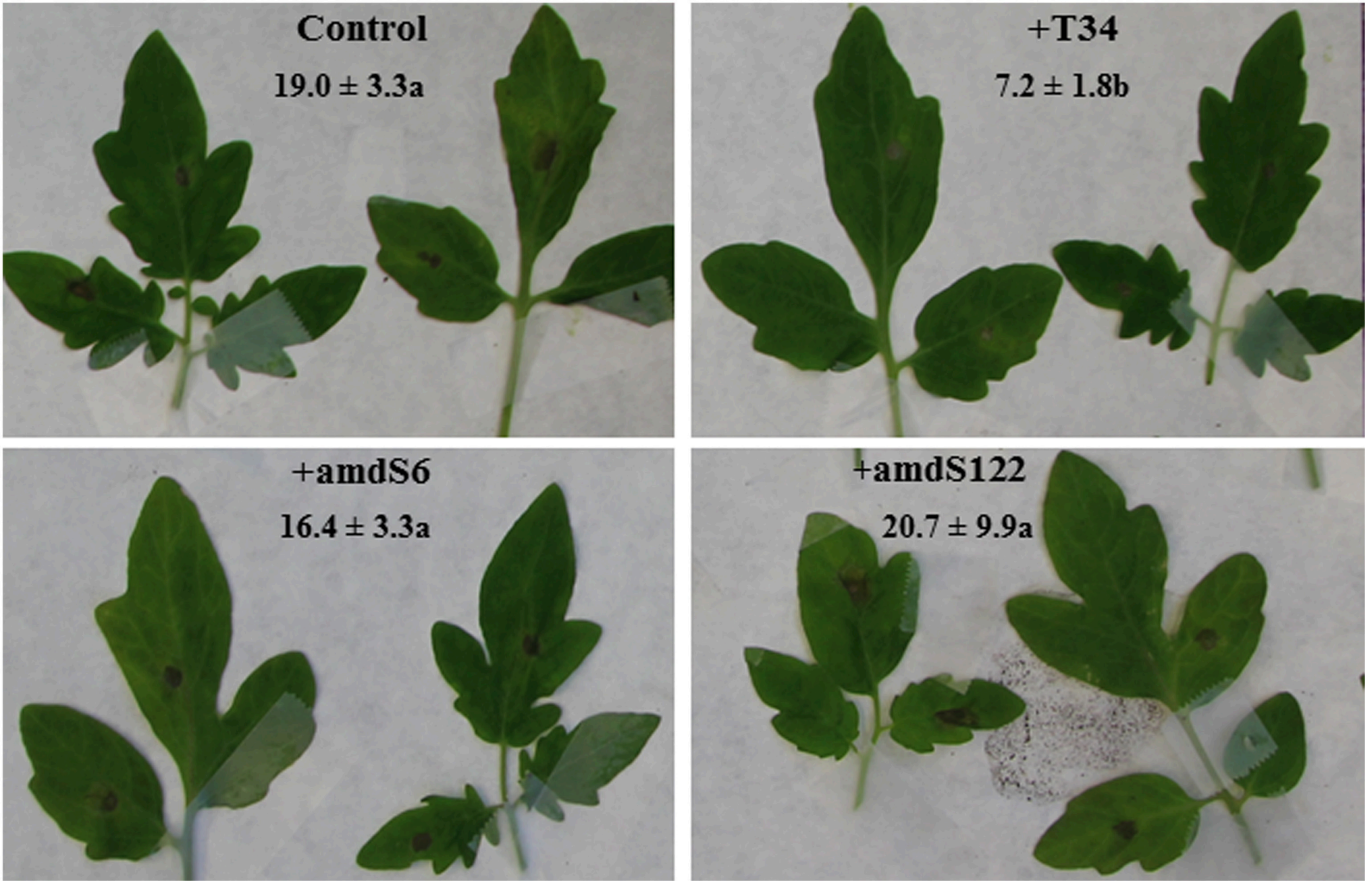
**Necrotic lesions observed in tomato leaves from *T. harzianum* seed treatment and *B. cinerea* conidia infection**. Untreated seed and *B. cinerea*-infected leaves (control) and *T. harzianum* T34-, transformants amdS6-, and amdS122-treated seed and *B. cinerea*-infected leaves. Three days after *B. cinerea*-infection, leaves were detached and photographed. Means of necrotic area percentages with standard deviations of six plants are indicated for every condition. The differences between each strain and the control were tested using one-way analysis of variance (ANOVA) followed by Tukey' s test. Values followed by different letters are significantly different (*P* < 0.05).

Results obtained in *B. cinerea*-infected tomato plants previously seed-coated by T34, amdS6, or amdS122 strains showed that *amdS* gene expression also affected the biocontrol activity of *T. harzianum* (Figure 3). The highest necrotic leaf areas were observed in the two *amdS* treatments, being similar to those of untreated plants.

In order to analyze the effect of the *amdS* gene expression in *T. harzianum* on the nutritional status of tomato plants treated with this fungus, we determined nitrogen and carbon levels in the aerial part of the control plants and plants treated with T34, amdS6, or amdS122 (Table 3). The three assayed *T. harzianum* strains were able to increase the levels of nitrogen and carbon in tomato plants, and higher amounts of these two elements were measured in plants previously seed-coated with *amdS* transformants than those observed in plants treated with T34. Similar results were obtained in two independent biological experiments.

### Transcriptional response of tomato plants to interaction with *T. harzianum* T34 and *amdS* transformants

**Figure 4:**
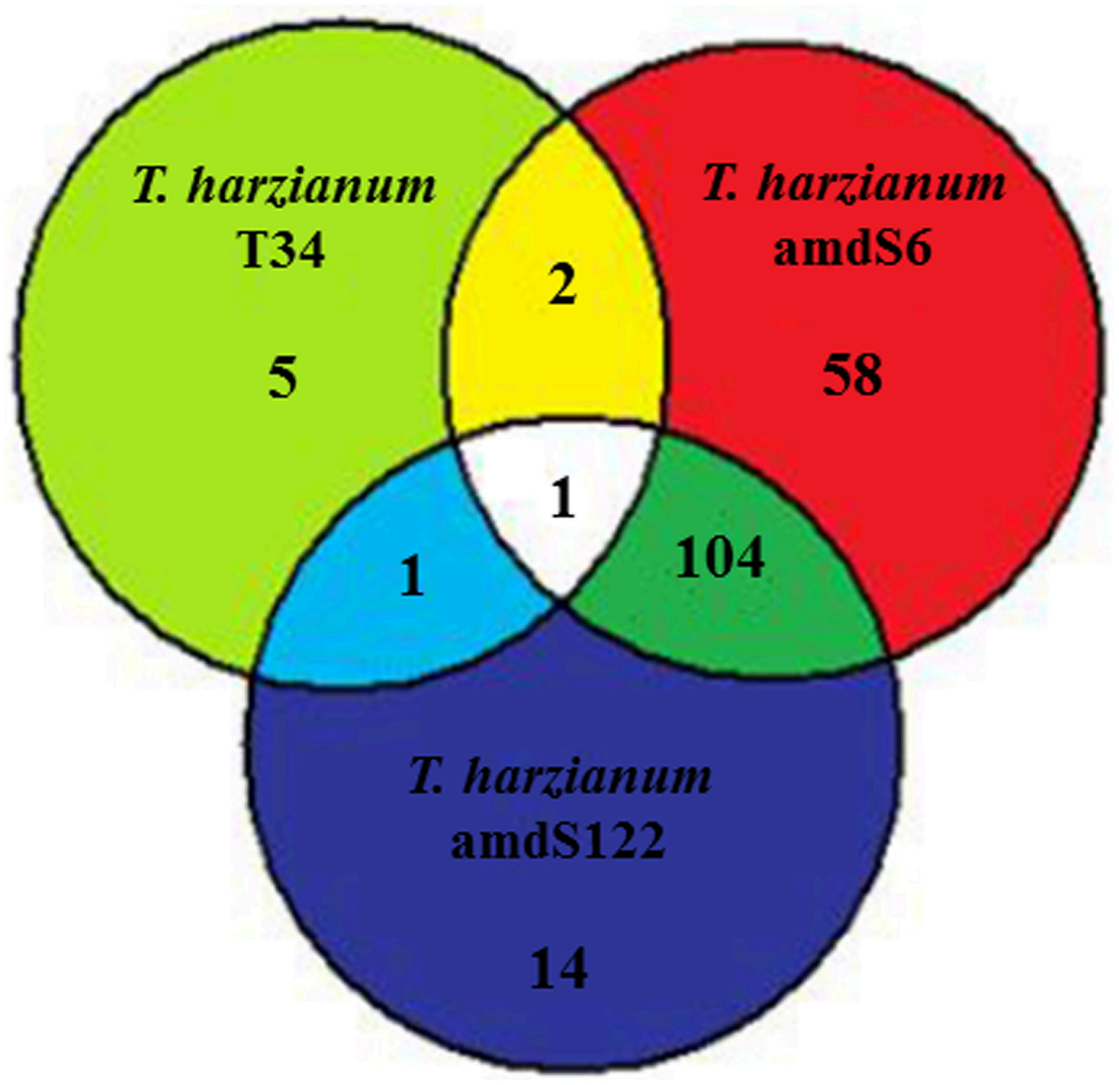
**Global expression data in tomato from microarray analysis**. Venn diagram representing the number of probe sets from tomato microarrays that showed significant changes in expression during interactions between tomato plants and *T. harzianum* wild-type (T34) or *amdS* transformants (amdS6 or amdS122) in comparison to control plants without *Trichoderma*.

A transcriptomic analysis using the Affymetrix GeneChip Tomato Genome Array was performed in order to ascertain the physiological and biochemical changes in 3-week-old tomato plants caused by treating the seeds with T34 or the *amdS* transformants. Of the total 10038 probe sets deposited on the microarray, representing 9254 genes, 185 (1.84%) showed at least a two-fold significant change (FDR 0.10) in expression in the presence of *T. harzianum* when compared with non-treated plants. The distribution of the 185 probe sets is shown in a Venn diagram (Figure 4).

A total of nine probe sets differed significantly (FDR 0.10) in expression by at least two-fold in the tomato plant-*T. harzianum* T34 interaction: six were upregulated, three of which showed homology with hypothetical proteins; and three were downregulated (Table 5 and Table S2). These probe sets were grouped into six different physiological processes. Except for the downregulation of a defensin gene, an increased expression of several genes encoding enzymes related to plant defense such as a threonine deaminase, a cathepsin D inhibitor, and a neryl diphosphate synthase 1 was observed. The decreased expression of genes encoding a phosphoenolpyruvate carboxykinase (PEPCK) and a non-symbiotic hemoglobin-1, related to carbohydrate metabolism and nitrogen assimilation, respectively was also observed.

**Table 5:** **Tomato probe sets differentially expressed in interaction with *T. harzianum* T34**.

**Physiological process**	**Hit description**	**Probe sets up**	**Probe sets down**
Carbohydrate metabolism	Phosphoenolpyruvate carboxykinase		1^*^
Amino acid metabolism	Threonine deaminase	1	
Secondary metabolism	Neryl diphosphate synthase 1	1^**^	
Nitrogen assimilation	Non-symbiotic hemoglobin-1		1^***^
Defense	Cathepsin D inhibitor	1^**^	
	Defensin		1
Unknown function		3	

*Summary of the probe sets expressed differentially (FC ≥ 2 and FDR 0.10) for tomato plants in interaction with T. harzianum T34 in comparison to control plants without Trichoderma. These probe sets were grouped into six different physiological processes, and their description was based on the homology with sequences of the UNIPROT database, using the BLAST algorithm and applying an E < 10^−10^ level*.

* *Probeset also expressed differentially (in this case upregulated) for tomato plants in interaction with amdS6 and amdS122 transformants*.

** *Probesets also expressed differentially (in this case upregulated) for tomato plants in interaction with amdS6 transformant*.

*** *Probeset also expressed differentially (in this case upregulated) for tomato plants in interaction with amdS122 transformant*.

We identified 105 probe sets that differed significantly (FDR 0.10) in expression by at least two-fold in both *amdS* transformant-tomato plant interactions: six were upregulated whereas 99 were downregulated (Table 6 and Table S3). These probe sets were grouped into several physiological processes, transcription and translation, signaling, carbohydrate metabolism, and defense processes being the most affected. Among six upregulated probe sets, four were related to carbohydrate metabolism [two phosphoenolpyruvate carboxylases (PEPC), a PEPCK, and one cellulose precursor] and one was a gene encoding a nitrate reductase (NR), related to nitrogen assimilation. However, nine genes corresponding to enzymes involved in carbohydrate metabolism such as phosphoglycerate mutase, dehydrogenase reductase, glycosyl transferase, endochitinase, xyloglucan endotransglycosylase, invertase, and acetylglucosaminidase, were downregulated.

**Table 6:** **Tomato probe sets differentially expressed in interaction with *T. harzianum amdS* transformants**.

**Physiological process**	**Hit description**	**Probe sets up**	**Probe sets down**
Carbohydrate metabolism	Phosphoenolpyruvate carboxylase	2	
	Phosphoenolpyruvate carboxykinase	1	
	Endo-1,4-β-glucanase	1	
	Phosphoglycerate mutase		1
	Short chain dehydrogenase/reductase		1
	Xyloglucan endo-transglycosylase		2
	Glycosyltransferase		1
	Acidic endochitinase		2
	Invertase		1
	Endo-β-N-acetylglucosaminidase		1
Lipid and fatty acid metabolism	Acyl-CoA synthetase		1
	Phospholipase		1
	3-ketoacyl-CoA thiolase		2
Amino acid metabolism	Prephenate dehydrogenase		2
	Arogenate dehydratase		1
	5-enolpyruvylshikimate-3-phosphate synthase		1
Carboxylic acid metabolism	Benzoil-CoA:benzyl alcohol benzoil transferase		1
Secondary metabolism	Tyramine hydroxycinnamoyl transferase		1
Energy metabolism	Alcohol dehydrogenase		1
Nitrogen assimilation	Nitrate reductase	1	
Signaling	Receptors		5
	Ubiquitin ligase		2
	Protein kinase		6
	DC1 domain binding protein		1
Transcription and translation (protein synthesis)	Transcriptional factor		14
	60s ribosomal protein		1
Hormonal response	Auxin response protein		2
	1-aminocyclopropane-1-carboxylate oxidase		1
	Abscisic acid response protein		1
Defense	NtEIG-E80 protein		1
	HSR203J protein		1
	Miraculin protein		1
	AAA ATPase		1
	*Verticillium* resistance protein		1
	ASC1 protein		1
	ATL2 protein		1
	*Phytophthora* inhibitor protease 1		1
	PR-5x related to pathogenesis protein		1
	Prolyl 4-hydroxylase		1
Transport	Metallic ions		1
	Hexose transporter		1
	Dicarboxylate transporter		1
	Lipid transporter		1
	Sodium-hydrogen exchange		1
	Protein transporter		1
Detoxification	Cytochrome p450 monooxygenase		2
	Glutathione S-transferase		4
Posttranslational events	Metalloprotease		1
	Aspartyl protease		1
Binding	Calcium binding protein		5
Cell wall and membranes	Methyl esterase inhibitor protein		1
Abiotic stress response	Cell wall peroxidase		1
	Dicyanin		1
	Nitrogen rich protein		1
	Heat shock protein		2
Unknown function		1	11

*Summary of the probe sets expressed differentially (FC ≥ 2 and FDR 0.10) in both amdS transformant-tomato plant interactions in comparison to control plants without Trichoderma. These probe sets were grouped into 18 different physiological processes, and their description was based on the homology with sequences of the UNIPROT database, using the BLAST algorithm and applying an E < 10^−10^ level*.

The relevance of transcription and translation processes was reflected by the downregulation of 15 probe sets, 14 of which corresponded to different types of transcriptional factors. Signaling processes were highlighted by the downregulation of five genes encoding kinase proteins, involved in responses to signals of different nature (i.e., light, hormones, temperature stress, nutrient starvation, or pathogen invasion), and four genes corresponding to membrane receptors and elicitor proteins, related to defense and disease resistance. Moreover, 12 genes related to plant defense responses against different pathogens [i.e., enhanced disease susceptibility 1 (*EDS1*), or a PR-5 encoding gene] were also downregulated. Genes involved in metabolism of lipids and fatty acids, amino acids, carboxylic acids, in the secondary and energetic metabolism, as well as in transport, detoxification, and posttranslational processes were also downregulated in 3-week-old tomato plants interacting with the *amdS* transformants.

According to the GO terminology, gene expression corresponding to biological processes in response to chemical stimuli (GO: 50896), in response to organic substances (GO: 10033), and defense response to stresses (GO: 6952) were statistically overrepresented in tomato plants colonized by the acetamidase mutants of *T. harzianum*. Plants challenged with amdS6 also showed statistically overrepresentation of cellular metabolic processes of aromatic (GO: 6725) and nitrogen (GO: 34641) compounds, compared to the control condition. As it might be expected, nitrogen metabolism is overrepresented in plants colonized by an acetamidase-expressing mutant.

To confirm the microarray results, quantitative real-time PCR was performed to analyze the expression of six genes: three upregulated (a PEPCK, an endo-1,4-β-glucanase, and a nitrate reductase) and three downregulated (a glycosyltransferase, a tyramine hydroxycinnamoyl transferase, and a phospholipase). Expression of these genes correlated with the microarray data (Figure S2).

## Discussion

Nitrogen is one of the most abundant elements on earth and is an essential nutrient for plants, being a major component of chlorophyll and amino acids. Nitrogen availability is a major limiting factor for growth, development, and productivity of crop plants because this element is often present in small quantities locally or is present in a form that cannot be used by the plant (Kraiser et al., 2011). In response to this problem, the evolution of many plant species has included the development of mutually beneficial symbiotic relationships with soil-borne microorganisms, which ensure the input of nitrogen to the plant. The inorganic nitrogen uptake in beneficial plant root-bacteria and -mycorrhiza interactions is well documented (Courty et al., 2015). In addition to the biocontrol action, beneficial effects of *Trichoderma* on plants have been reported in terms of growth promotion and defense induction against biotic and abiotic stresses (Hermosa et al., 2012; Rubio et al., 2014). Thus, plants treated with *Trichoderma* spp. may be larger and healthier and have greater yields than untreated plants (Shoresh et al., 2010). Although, studies regarding the role of *Trichoderma* in the symbiotic nitrogen assimilation, as a major component of such beneficial responses, are still scarce, it was reported in the late 1990s that *T. harzianum* favored nitrogen uptake in maize field trials (Harman, 2000). Moreover, the present study has aimed to provide additional evidence of this behavior at a molecular level.

Tomato plants from seeds coated with *T. harzianum* transformants carrying a disruption cassette that contain the *A. nidulans amdS* gene showed an increase in root length, much more evident than in plants developed from seeds coated with the wild-type strain. This result suggested that this selectable marker gene was not completely innocuous for the host strain. This observation led us to obtain *T. harzianum* T34 transformants expressing the *amdS* gene and permitted the analysis of the role of acetamidase activity in the interaction of this fungus with plants. The *amdS* transformants amdS6 and amdS122 were selected since they exhibited one and two copies of the transformation cassette, respectively, in a Southern blot analysis. We used two culture media to compare *amdS* gene expression and ammonium production between T34 and these two *amdS* transformants. The *amdS* expression levels detected in PDB and PDB plus acetamide cultures are in agreement with the copy number of the transformation cassette inserted in each transformant (Figure 1) but are independent of the culture medium used for fungal growth, as expected for a gene expressed under a constitutive promoter. Since the *amdS* gene of *A. nidulans* is able to release acetate and ammonium from aliphatic amides (Hynes and Pateman, 1970; Chacko et al., 2009), as is the case of acetamide, ammonium levels were only detected in *amdS* transformant supernatants from cultures containing this substrate. Our results correlate with the absence of *amdS* orthologous genes in the *Trichoderma* spp. genomes. The *amdS* heterologous expression in *T. harzianum* did not affect the growth of this fungus in media without acetamide (Table 1) but did confer the use of this amide as a nitrogen source. In fact, this feature has allowed *amdS* to be used as a selectable marker for the transformation of filamentous fungi, including *Trichoderma* spp. (Penttilä et al., 1987; Rodríguez and Yoder, 1987; Gruber et al., 2012).

As no significant growth differences were detected among *T. harzianum* strains in several culture media without acetamide and a similar antifungal behavior was observed for T34 and *amdS* transformants in dual cultures, it might be thought that the acetamidase activity of *T. harzianum* is not contributing to the biocontrol potential of this fungus. However, as observed in PDA membrane assays, the significantly higher antifungal activity of *amdS* transformants against *F. oxysporum* compared to that of wild-type may indicate that acetamidase activity modifies the culture medium composition or contributes to the production of a pool of secondary metabolites that would be involved in the inhibition of this pathogen. In any case, no differences in the antifungal activity of the *T. harzianum* strains against the two pathogens assayed, *F. oxysporum* and *B. cinerea* were observed in the tests performed with 48 h-PDB supernatants (Figure 2). As shown in Table 2, the lower *F. oxysporum* and *B. cinerea* growth inhibition values recorded for *amdS* transformants compared to those of the wild-type in the two PDA plus acetamide membrane assays must not be associated to a reduced antifungal activity of these two transformants. Thus, the previous growth of amdS6 or amdS122 would reduce acetamide concentration in the culture medium, this decrease would in fact facilitate the subsequent growth of the pathogens.

As in many previous studies, where the ability of *Trichoderma* spp. to promote plant growth has been described (Shoresh et al., 2010; Hermosa et al., 2012), T34 inoculation significantly increased the stem length of plants compared to the uninoculated control. In our assays, *in vitro* plants grown in the presence of *amdS* transformants were significantly longer than those grown in presence of the wild-type strain. This observation may be explained by the greater availability of nutrients (i.e., nitrogen sources) in plants confronted with strains displaying amidase activity, as no growth differences were observed among *T. harzianum* strains grown on the complex medium MS. Results from greenhouse assays showed that the three *T. harzianum* strains significantly increased the stem and root lengths of tomato plants, but to a greater extent in the *amdS* transformant treatments, and also confirmed the radical plasticity model that relates high concentrations of ammonium in the medium to an increased growth of the taproot (Zhang and Forde, 2000). Since plants treated with the *amdS* transformants displayed the highest nitrogen and carbon levels, they would be good candidates as plant enhancers. It has been reported that *Trichoderma* spp. can favor nutrient uptake in plants and such increase can substantially improve crop yields (Harman, 2000; Shoresh et al., 2010). However, plants treated with *amdS* transformants showed similar level of defense against *B. cinerea* than control plants but also exhibited an increased susceptibility to this pathogen in comparison with the response observed in plants challenged with T34.

The transcriptomic analysis of *T. harzianum* seed-coated tomato plants at 3 weeks after fungal application allowed us to identify significant transcriptional changes in the host plant elicited by strain T34 and the transformants amdS6 and amdS122. The parameters FC ≥ 2 and FDR 0.10, used to analyze microarray data, can be considered adequate since these limits have been used in previous transcriptomic studies performed in tomato (Ruzicka et al., 2010; Shi et al., 2013). Previous transcriptomic studies have shown that after 24 h of *Arabidopsis* root-inoculation by *T. harzianum* T34, expression changes in genes related to plant responses to both biotic and abiotic stress conditions were detected in aerial parts of *Arabidopsis* plants. These changes involved several plant signal transduction pathways controlled by phytohormones such as SA, JA, auxins, or abscisic acid, indicating an important transcriptomic activity at early interaction times (Morán-Diez et al., 2009, 2012). Although direct comparisons cannot be made due to the different plant species, the fungal application method and the distinct interaction times that were used in the present study, it was still unexpected that only nine genes would be differentially expressed in the *T. harzianum* T34-tomato interaction with uninoculated tomato plants. This could be explained by the fact that after 3 weeks, when the root colonization is well established, changes in plant shoot transcriptome were not so drastic as in early colonization times.

Among these nine genes, the six upregulated encode proteins related to plant defense responses or increase their expression under biotic stress conditions. The three downregulated genes were the typical type of marker gene for the JA-defense cascade *PDF1.2* (Thomma et al., 2002); the non-symbiotic class 1 hemoglobin gene, involved in nitrogen metabolism modulation and strongly induced by nitrate, nitrite, and nitric oxide (Ohwaki et al., 2005) also related to the plant mineral nutrition and nitrogen assimilation (Wang et al., 2003); and a gene encoding a PEPCK involved in carbon metabolism with several functions such as the atmospheric CO_2_ assimilation or the reposition of the consumed TCA intermediates in the biosynthesis and assimilation of nitrogen (O'Leary et al., 2011).

The majority (94%) of genes that differed significantly in expression in both *amdS* transformant-tomato plant interactions was downregulated (Table 6 and Table S3). This highlighted those genes related to plant defense responses and disease resistance that correlated with the larger necrotic leaf areas produced by *B. cinerea* in plants treated with *amdS* transformants in comparison to those observed in plants treated with the wild-type.

Among the few upregulated genes in *amdS* transformant-tomato plant interactions, those encoding PEPC, PEPCK, and NR, which co-ordinate primary nitrogen and carbon assimilation in leaves (Foyer et al., 2003), relate the increase in ammonium supply favored by the acetamidase activity of strains amdS6 and amdS122 with enhanced plant nitrogen uptake and photosynthetic rates. Interestingly, PEPCK gene was downregulated in plants treated with T34, as indicated above. PEPCK is associated with plant tissues in which the metabolism of nitrogenous compounds is enhanced (Delgado-Alvarado et al., 2007) and its upregulation in tomato plants colonized by *amdS* transformants is in agreement with the increased ammonium availability due to *Trichoderma* strains displaying acetamidase activity. We have detected the highest carbon and nitrogen levels in plants treated with *amdS* transformants. The link between carbon and nitrogen is critical and unless there is sufficient carbon available, improving a plant' s ability to take up and utilize nitrogen may be compromised (McAllister et al., 2012). It has been shown that nitrogen levels can significantly affect carbon fixation (Reich et al., 2006). Nitrogen is stored in large quantities in photosynthetic proteins, such as rubisco and PEPC, and decreases in nitrogen assimilation and storage will thus decrease the overall amount of carbon fixed by the plant (Nunes-Nesi et al., 2010). When PEPC was investigated in wheat, barley, and tomato roots fed with different nitrogen sources, ammonium-fed plants exhibited a 2–2.5-fold higher PEPC activity than nitrate-fed plants at 7 days after the onset of the nitrogen supply (Koga and Ikeda, 1997). NR catalyzes the reduction of nitrate to nitrite, and beneficial fungi such as *Piriformospora indica* or *Trichoderma* spp. increase the expression of genes encoding NR in plants (Sherameti et al., 2005; Bae et al., 2009) and hence may be involved in nitrogen accumulation through the symbiotic association leading to efficient nitrogen use and increased development. Nitrite is highly toxic to plant cells and is reduced to ammonium by the enzyme nitrite reductase. This enzyme was also upregulated in both *amdS* transformant-tomato plant interactions, but with a FC < 2 (1.78) in the interaction with amdS6, therefore it does not appear reflected in the Venn diagram.

The downregulation of defense genes and the upregulation of carbon and nitrogen metabolism genes observed in the microarrays, the higher sensitivity to *B. cinerea* infections, and the increased growth and carbon and nitrogen levels observed in plants treated with *amdS* transformants in greenhouse assays suggest that the positive effects in tomato plant growth caused by the *Trichoderma* acetamidase activity were accompanied by an increased sensitivity to a necrotrophic pathogen. Although, studies highlighting the role of *Trichoderma* in the symbiotic nitrogen uptake and its efficient use as a major component of the beneficial responses of plants are still scarce, the present study has tried to provide further confirmation of this behavior at a molecular level.

## Author contributions

SD and MR performed the experiments; SD, MR, and EM conceived and designed the experiments; SD, MR, RH, and EM analyzed the data; CN, WB, RC, and SG contributed reagents/materials/analysis tools; MR, RH, CN, and EM wrote the paper.

### Conflict of interest statement

The authors declare that the research was conducted in the absence of any commercial or financial relationships that could be construed as a potential conflict of interest.
