# Supplementary material for: Nitrogen Metabolism and Growth Enhancement in Tomato Plants Challenged with Trichoderma harzianum Expressing the Aspergillus nidulans Acetamidase amdS Gene
Source: Front Microbiol. 2016 Aug 3;7:1182. doi: 10.3389/fmicb.2016.01182 (PMC4971021; doi:10.3389/fmicb.2016.01182)
Supplement: Supplementary file 1 [file Table1.DOCX]

**TABLE S1. Primers used in the present study.**

| **Primer** | **Target gene** | **Sequence (5’-3’)** | **Slope** | **Efficiency** |
| --- | --- | --- | --- | --- |
| **For gene isolation** |  |  |  |  |
| amdS-NT | *amdS* | ATGCCTCAATCCTGGGAA |  |  |
| amdS-CT |  | CTATGGAGTCACCACATT |  |  |
| **For screening of transformant strains** |  |  |  |  |
| amdS-3 |  | TTCCTTCCCGGCCTTTTCTT |  |  |
| gpd3F |  | ATCTTCAGTATATTCATCTTCCCATC |  |  |
| cbh2 |  | GTTGCTCATTTGCGGTCTACC |  |  |
| **For Real-time PCR** |  |  |  |  |
| amdS-RT1 | *amdS* | TCGACGGCAATGTCCTTCCAC | -3.29 | 101.38 |
| amdS-RT2 |  | TCGCCGGATGCACTGATATC |  |  |
| Act-1-tricho | *actin* | ATCGGTATGGGTCAGAAGGA | -3.41 | 96.33 |
| Act-2-tricho |  | ATGTCAACACGAGCAATGG |  |  |
| PCK-fw | Phosphoenolpyruvate carboxykinase | CTCAGAGGGAATTTGAGGTTTCC | -3.09 | 110.77 |
| PCK-rev |  | GTGTCTCAGTACTTGTTCAGCTG |  |  |
| BGP-fw | Endo-1,4-β-glucanase | GAGAGGAGATTCCGCACTCATC | -3.24 | 103.63 |
| BGP-rev |  | AGCAGCCCAACTCAACAAGGTT |  |  |
| NR-fw | Nitrate reductase | GTACGAGCTATCGATGAGACCC | -3.24 | 103.20 |
| NR-rev |  | ATTGGTTTCCAGGTTGGGTC |  |  |
| HTTP-fw | Tyramine hydroxycinnamoyl transferase | ATGGCTCCTGCTCTTGAACAA | -3.01 | 114.77 |
| HTTP-rev |  | ATCAGATTTCGTAGCGAGACG |  |  |
| PLDB1-fw | Phospholipase | TCCTGATATTTTGGGAATAGC | -3.11 | 107.99 |
| PLDB1-rev |  | CAGCACATTCTTACCACACACTA |  |  |
| Avr9N-fw | Glycosyltransferase | GGTGGAGATAATTTGGTGAATA | -3.04 | 113.51 |
| Avr9N-rev |  | TTATGATTTGTATAGATTGAGCC |  |  |
| Act-F-tomato | *actin* | ATCGGTATGGGTCAGAAGGA | -3.35 | 98.89 |
| Act-R-tomato |  | ATGTCAACACGAGCAATGG |  |  |
