# Supplementary material for: Nitrogen Metabolism and Growth Enhancement in Tomato Plants Challenged with Trichoderma harzianum Expressing the Aspergillus nidulans Acetamidase amdS Gene
Source: Front Microbiol. 2016 Aug 3;7:1182. doi: 10.3389/fmicb.2016.01182 (PMC4971021; doi:10.3389/fmicb.2016.01182)
Supplement: Supplementary file 2 [file Table2.DOCX]

**Table S2.** Probe sets expressed differentially (FC ≥ 2 and FDR 0.10) for tomato plants in interaction with *T. harzianum* T34 in comparison to control plants without *Trichoderma*. These probe sets were grouped into six different physiological processes, and their description was based on the homology with sequences of the UNIPROT database, using the BLAST algorithm and applying an E-value < 10^-10^ level.

| **Upregulated** | | | |
| --- | --- | --- | --- |
| **Physiological process** | **Hit description** | **FC** | **Probe ID in Affimetrix genome array** |
| Amino acid metabolism | Threonine deaminase | +2.52 | Les.4488.1.S1_at |
| Secondary metabolism | Neryl diphosphate synthase 1 | +2.44 | Les.415.1.A1_a_at* |
| Defense | Cathepsin D inhibitor | +2.01 | Les.1783.1.A1_at* |
| Unknown function |  | +2.86  +2.02  +2.00 | Les.1077.1.S1_at  Les.2839.1.S1_at  Les.2460.2.S1_at |
| **Downregulated** | | | |
| **Physiological process** | **Hit description** | **FC** | **Probe ID in Affimetrix genome array** |
| Carbohydrate metabolism | Phosphoenolpyruvate carboxykinase | -2.53 | Les.3539.1.S1_at^**^ |
| Nitrogen assimilation | Non-symbiotic haemoglobin-1 | -2.17 | Les.3700.1.S1_at^***^ |
| Defense | Defensin | -2.84 | Les.3983.1.S1_at |

^*^Probesets expressed also differentially for tomato plants in interaction with amdS6 transformant, with FC of +3.27 (Les.415.1.A1_a_at) and +2.02 (Les.1783.1.A1_at).

^**^Probeset expressed also differentially for tomato plants in interaction with amdS6 and amdS122 transformants, with FC of +2.38 and +4.29, respectively.

^***^Probeset expressed also differentially for tomato plants in interaction with amdS122 transformant, with a FC of +3.39.
