# Supplementary material for: Nitrogen Metabolism and Growth Enhancement in Tomato Plants Challenged with Trichoderma harzianum Expressing the Aspergillus nidulans Acetamidase amdS Gene
Source: Front Microbiol. 2016 Aug 3;7:1182. doi: 10.3389/fmicb.2016.01182 (PMC4971021; doi:10.3389/fmicb.2016.01182)
Supplement: Supplementary file 3 [file Table3.DOCX]

**Table S3.** Probe sets expressed differentially (FC ≥ 2 and FDR 0.10) in both amdS transformant-tomato plant interactions in comparison to control plants without *Trichoderma*. These 105 probe sets, 6 upregulated and 99 downregulated, were grouped into 18 different physiological processes, and their description was based on the homology with sequences of the UNIPROT database, using the BLAST algorithm and applying an E-value < 10^-10^ level.

| **Upregulated** | | | |
| --- | --- | --- | --- |
| **Physiological process** | **Hit description** | **FC (amdS6/amdS122)** | **Probe ID in Affimetrix genome array** |
| Carbohydrate metabolism | Phosphoenolpyruvate carboxylase | +2.18/+4.12  +2.08/+3.66 | Les.2909.2.S1_at  Les.2909.1.S1_at |
|  | Phosphoenolpyruvate carboxykinase | +2.38/+4.29 | Les.3539.1.S1_at* |
|  | Endo-1,4-β-glucanase | +2.21/+2.84 | Les.3667.1.S1_at |
| Nitrogen assimilation | Nitrate reductase | +2.01/2.65 | LesAffx.45315.4.S1_at |
| Unknown function |  | +2.31/+2.59 | LesAffx.48076.1.S1_at |
| **Downregulated** | | | |
| **Physiological process** | **Hit description** | **Fold change** | **Probe ID in Affimetrix genome array** |
| Carbohydrate metabolism | Phosphoglycerate mutase | -6.21/-9.79 | Les.5567.1.S1_at |
|  | Short chain dehydrogenase/reductase | -3.56/-5.22 | Les.5930.1.S1_at |
|  | Xyloglucan endo-transglycosylase | -3.54/-4.23  -4.39/-4.02 | Les.429.1.S1_at  Les.4353.1.S1_at |
|  | Glycosyltransferase | -2.46/-4.08 | Les.3777.1.S1_at |
|  | Acidic endochitinase | -4.13/-4.01  -3.88/-3.38 | Les.435.1.S1_at  LesAffx.69659.1.S1_at |
|  | Invertase | -4.34/-3.80 | Les.3460.1.S1_at |
|  | Endo-β-N-acetylglucosaminidase | -2.04/-2.50 | Les.5443.1.S1_at |
| Lipid and fatty acid metabolism | Acyl-CoA synthetase | -7.48/-10.68 | LesAffx.10235.1.S1_at |
|  | Phospholipase | -6.12/-8.49 | Les.3493.1.S1_at |
|  | 3-ketoacyl-CoA thiolase | -2.49/-3.45  -2.53/-3.20 | Les.2747.1.S1_at  Les.2747.2.S1_at |
| Amino acid metabolism | Prephenate dehydrogenase | -7.46/-6.68  -2.60/-2.70 | LesAffx.66354.1.S1_at  Les.5555.1.S1_at |
|  | Arogenate dehydratase | -2.16/-3.18 | LesAffx.10955.3.S1_at |
|  | 5-enolpyruvylshikimate-3-phosphate synthase | -2.37/-2.88 | Les.5458.1.S1_at |
| Carboxylic acid metabolism | Benzoil-CoA:benzyl alcohol benzoil transferase | -3.44/-3.13 | Les.2529.2.S1_at |
| Secondary metabolism | Tyramine hydroxycinnamoyl transferase | -3.61/-4.39 | Les.4038.1.S1_at |
| Energy metabolism | Alcohol dehydrogenase | -2.28/-2.89 | Les.3418.3.S1_at |
| Signaling | Receptors | -7.53/-12.30  -6.96/-8.54  -3.05/-4.44  -3.03/-3.84  -3.01/-3.73 | Les.5044.1.S1_at  Les.2137.1.S1_at  Les.5208.1.S1_at  LesAffx.68107.1.S1_at  Les.5939.1.S1_at |
|  | Ubiquitin ligase | -6.86/-5.69  -3.19/-4.74 | LesAffx.22812.2.S1_at  LesAffx.30683.1.S1_at |
|  | Protein kinase | -3.83/-4.26  -2.50/-3.37  -3.09/-3.50  -2.39/-3.07  -2.83/-3.00  -2.20/-2.70 | LesAffx.10313.1.A1_at  LesAffx.70335.1.S1_at  Les.5205.1.S1_at  Les.4316.1.S1_at  Les.1806.1.S1_at  LesAffx.16424.1.S1_s_at |
|  | DC1 domain binding protein | -3.68/-3.37 | LesAffx.51266.1.S1_at |
| Transcription and translation (protein synthesis) | Transcriptional factors | -11.37/-10.89  -4.03/-8.43  -3.62/-7.70  -3.36/-6.88  -3.70/-4.80  -5.81/-4.79  -3.95/-4.65  -3.10/-4.46  -2.95/-4.23  -5.18/-4.02  -6.54/-4.00  -2.25/-3.76  -2.46/-3.67  -2.62/-3.40 | Les.3575.1.S1_at  LesAffx.63523.2.S1_at  Les.5699.1.S1_at  Les.4483.1.S1_at  Les.3964.1.S1_at  LesAffx.71311.1.S1_at  LesAffx.837.1.S1_at  LesAffx.4793.1.S1_at  LesAffx.43341.1.S1_at  Les.3551.1.S1_at  LesAffx.63523.1.S1_at  LesAffx.64439.1.S1_at  LesAffx.36712.1.S1_at  LesAffx.56634.1.S1_at |
|  | 60s ribosomal protein | -4.70/-8.81 | LesAffx.71577.1.S1_a_at |
| Hormonal response | Auxin response protein | -2.57/-7.32  -3.12/-3.06 | Les.3486.1.S1_at  LesAffx.3081.1.S1_at |
|  | 1-aminocyclopropane-1-carboxylate oxidase | -4.96/-5.20 | Les.2560.1.S1_at |
|  | Abscisic acid response protein | -2.50/-2.63 | Les.4807.1.S1_at |
| Defense | NtEIG-E80 protein | -13.29/-14.43 | LesAffx.344.3.S1_at |
|  | HSR203J protein | -8.84/-13.28 | LesAffx.18587.1.S1_at |
|  | Miraculin protein | -5.36/-11.77 | Les.5884.1.S1_at |
|  | AAA ATPase | -7.61/-8.50 | LesAffx.16102.1.S1_at |
|  | *Verticillium* resistance protein | -6.75/-6.10 | Les.3505.1.S1_at |
|  | ASC1 protein | -2.90/-4.48 | Les.3710.1.S1_at |
|  | ATL2 protein | -2.68/-4.31 | Les.5654.1.S1_at |
|  | *Phytophthora* inhibitor protease 1 | -4.48/-4.07 | LesAffx.11941.1.S1_at |
|  | PR-5x related to pathogenesis protein | -3.38/-2.99 | Les.3683.1.S1_at |
|  | Prolyl 4-hydroxylase | -2.60/-2.79 | LesAffx.50540.1.S1_at |
| Transport | Metallic ions | -8.34/-12.19 | LesAffx.51300.1.S1_at |
|  | Hexose transporter | -6.54/-6.26 | Les.3774.1.S1_at |
|  | Dicarboxylate transporter | -3.59/-4.50 | Les.4779.1.S1_at |
|  | Lipid transporter | -5.21/-3.85 | Les.5759.1.S1_at |
|  | Sodium-hydrogen exchange | -3.15/-3.84 | Les.1806.2.A1_at |
|  | Protein transporter | -2.39/-3.01 | Les.5755.1.S1_at |
| Detoxification | Cytochrome p450 monooxygenase | -11.51/-10.42  -7.56/-8.21 | Les.4880.1.S1_at  LesAffx.9038.3.S1_at |
|  | Glutathione S-transferase | -8.22/-9.11  -3.88/-3.89  -2.68/-2.77  -3.12/-2.64 | Les.2544.1.A1_at  LesAffx.3002.1.S1_at  Les.123.1.S1_at  Les.4501.1.S1_at |
| Posttranslational events | Metalloprotease | -2.65/-3.76 | LesAffx.63935.1.S1_at |
|  | Aspartyl protease | -2.44/-2.75 | Les.5217.1.S1_at |
| Binding | Calcium binding protein | -13.49/-16.04  -7.42/-9.36  -6.51/-6.37  -4.43/-5.49  -4.01/-4.44 | LesAffx.16164.1.S1_at  Les.1997.1.S1_at  Les.1997.3.A1_at  LesAffx.11542.1.S1_at  LesAffx.66814.1.S1_at |
| Cell wall and membranes | Methyl esterase inhibitor protein | -2.44/-3.84 | Les.4287.1.S1_at |
| Abiotic stress response | Cell wall peroxidase | -3.95/-7.37 | LesAffx.57363.1.S1_at |
|  | Dicyanin | -3.07/-3.17 | Les.217.1.S1_at |
|  | Nitrogen rich protein | -2.18/-3.16 | Les.5131.1.S1_at |
|  | Heat shock protein | -3.81/-2.89  -2.58/-2.83 | Les.3578.1.S1_at  Les.4819.1.S1_at |
| Unknown function |  | -2.22/-2.88  -2.66/-4.67  -3.73/-4.04  -2.92/-4.18  -2.73/-3.66  -5.12/-6.81  -4.24/-6.27  -2.77/-3.54  -3.00/-3.07  -2.73/-4.13  -2.88/-3.79 | LesAffx.44417.1.S1_at  Les.5090.1.S1_at  LesAffx.57303.1.S1_at  LesAffx.17017.1.S1_at  Les.2738.1.S1_at  Les.2529.1.A1_at  Les.5933.1.S1_at  Les.4222.1.S1_at  LesAffx.57779.1.S1_at  LesAffx.64990.1.S1_at  LesAffx.30683.2.S1_at |

^*^Probeset also expressed differentially for tomato plants in interaction with T34, with a FC of -2.53.
