# Supplementary figures and images for: Nitrogen Metabolism and Growth Enhancement in Tomato Plants Challenged with Trichoderma harzianum Expressing the Aspergillus nidulans Acetamidase amdS Gene
Source: Front Microbiol. 2016 Aug 3;7:1182. doi: 10.3389/fmicb.2016.01182 (PMC4971021; doi:10.3389/fmicb.2016.01182)

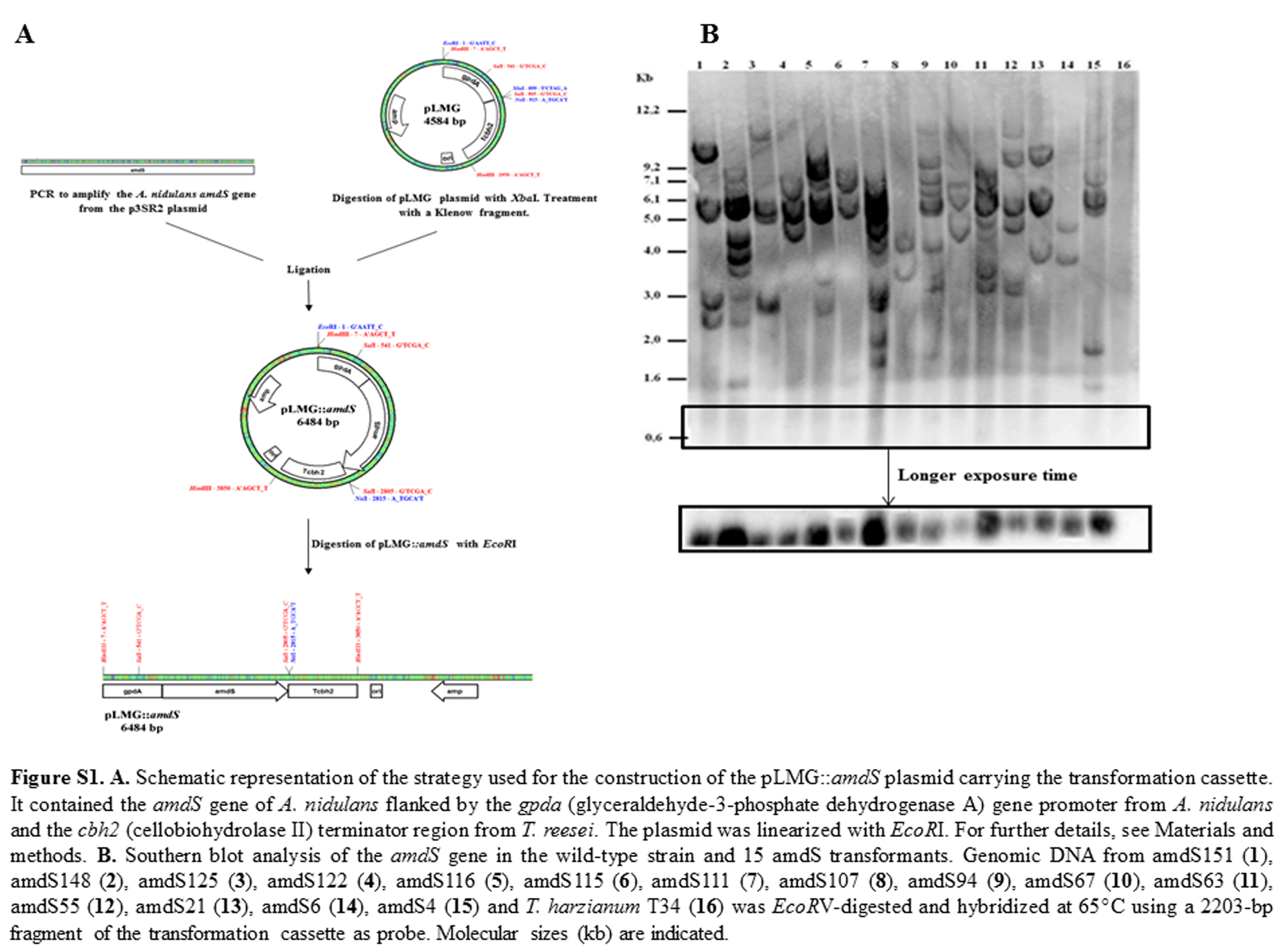

Supplement: Supplementary file 4 [file Image1.TIF]
