# Supplementary material for: Nitrogen Metabolism and Growth Enhancement in Tomato Plants Challenged with Trichoderma harzianum Expressing the Aspergillus nidulans Acetamidase amdS Gene
Source: Front Microbiol. 2016 Aug 3;7:1182. doi: 10.3389/fmicb.2016.01182 (PMC4971021; doi:10.3389/fmicb.2016.01182)
Supplement: Supplementary file 5 [file Image2.PDF]

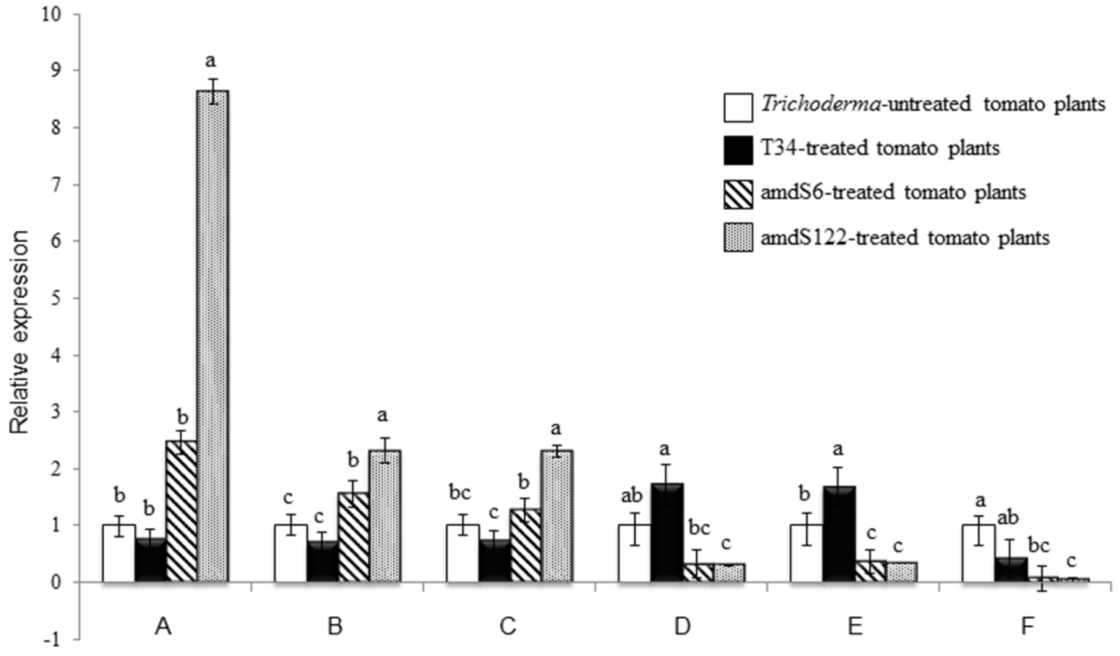

Figure S2. Validation of microarray experiments. Real-time PCR of a phosphoenolpyruvate carboxykinase (AAF19403) (A), an endo-1,4-β-glucanase (AAA69908) (B), a nitrate reductase (P17570) (C), a glycosyl transferase (AAG43554) (D), a tyramine hydroxycinnamoyl transferase (AAL99189) (E) and a phospholipase (AAG45487) (F) genes in 3-week-old tomato plants developed from *Trichoderma*-untreated, T34-treated, amdS6-treated or amdS122-treated seeds. Values correspond to relative measurements against tomato plants developed from *Trichoderma*-untreated seeds ($2^{-\Delta\Delta Ct} = 1$). The tomato *actin* gene was used as an internal reference gene. Bars represent standard deviations of the mean values of three biological replicates. The levels of expression were tested using one-way analysis of variance (ANOVA) followed by Tukey´s test. Each gene was analyzed independently and different letters represent significative differences ($P < 0.05$).
